# Functional Responses of Retaliatory Killing versus Recreational Sport Hunting of Leopards in South Africa

**DOI:** 10.1371/journal.pone.0125539

**Published:** 2015-04-23

**Authors:** Lourens H. Swanepoel, Michael J. Somers, Fredrik Dalerum

**Affiliations:** 1 Department of Zoology, University of Venda, Private bag X5050, Thohoyandou, 0950, South Africa; 2 Centre for Wildlife Management, Hatfield Experimental Farm, University of Pretoria, Private bag X20, Pretoria, 0028, South Africa; 3 Centre for Invasive Biology, University of Pretoria, Private bag X20, Pretoria, 0028, South Africa; 4 Mammal Research Institute, Department of Zoology and Entomology, University of Pretoria, Private bag X20, Pretoria, 0028, South Africa; University of Western Ontario, CANADA

## Abstract

Predation strategies in response to altering prey abundances can dramatically influence the demographic effects of predation. Despite this, predation strategies of humans are rarely incorporated into quantitative assessments of the demographic impacts of humans killing carnivores. This scarcity largely seems to be caused by a lack of data. In this study, we contrasted predation strategies exhibited by people involved in retaliatory killing and recreational sport hunting of leopards (*Panthera pardus*) in the Waterberg District Municipality, South Africa. We predicted a specialist predation strategy exemplified by a type II functional response for retaliatory killing, and a generalist strategy exemplified by a type III functional response for recreational sport hunting. We could not distinguish between a type I, a type II, or a type III functional response for retaliatory killing, but the most parsimonious model for recreational sport hunting corresponded to a type I functional response. Kill rates were consistently higher for retaliatory killing than for recreational sport hunting. Our results indicate that retaliatory killing of leopards may have severe demographic consequences for leopard populations, whereas the demographic consequences of recreational sport hunting likely are less dramatic.

## Introduction

Large carnivores are important regulatory components of terrestrial ecosystems [1]. However, large carnivores are also extinction prone [2] and predisposed to human wildlife conflict, which often leads to retaliatory killing [3]. In addition, hunting is commonly used as a tool to increase stakeholder incentives for large carnivore conservation [4]. Both hunting [5,6] and retaliatory killing [3,7] can produce strong direct and indirect effects on carnivore populations. Subsequently, numerous studies have used quantitative models to predict the demographic consequences of different mortality regimes in carnivore populations [8,9]. However, models evaluating the impacts of harvest or other human caused mortality are still hampered by a lack of information regarding the responses of people to altering carnivore abundance [10].

Predation strategies can broadly be classified along a gradient from specialists to generalists [11,12]. The functional response, i.e. the relationship between the consumption rate of a predator and the abundance of its prey, are often used to define predation strategies [11]. Specialist species are characterized by a type II functional response, in which predation rate increases at low levels of prey abundance until an upper asymptote is reached [11,13,14]. Generalist predators are exemplified by a type III functional response, which is characterized by a sigmoid relationship where prey abundance needs to rise above a critical threshold before predation rate increases [11,14,15]. A linear relationship between prey abundance and predation rate, i.e. a type I functional response, is rarely found among vertebrates [16]. Such a relationship can indicate an opportunistic strategy, since predation occurs at random every time a prey item is encountered [13]. Predation by specialist predators is believed to have destabilizing effects on prey populations because high predation rates are maintained even at low prey abundances [11,12]. Predation by generalist predators, on the other hand, is thought to have stabilizing effects on prey populations because these are relieved from predation pressure at low abundances [12,17]. Predation from opportunistic predators, exhibiting linear relationships between predation rate and prey abundance, have been considered to have partially stabilizing effects relative to predation from specialist predators [18].

The predatory response exhibited by humans to varying carnivore abundances likely depends on the type of interaction between humans and carnivores. For example, we could predict that humans engaged in retaliatory killing would respond differently to varying carnivore abundance than humans engaged in recreational sport hunting because of the different motivational drivers behind the two activities [10]. Retaliatory killing of carnivores is typically motivated by financial losses inflicted by predation, in combination with social, psychological and cultural factors [3,19,20]. Land users are typically resident, so that the cost of predation can be high for a specific land user even at very low carnivore abundances (e.g. leopard *Panthera pardus*) predation on financially valuable game [3,21]). Therefore, we suggest that humans engaged in retaliatory killing of problem animals will behave like a specialist predator, and continue to kill carnivores even at very low abundances. Recreational sport hunters of large carnivores, on the other hand, are typically motivated by the size of the trophy, the rarity of the target animal or the charisma effect [22,23]. Recreational sport hunters are limited by hunting time and are normally not resident in the area they hunt in. Therefore, we hypothesise that at some threshold of carnivore density, recreational sport hunters should abandon hunting and therefore behave like a generalist predator [24].

In this study, we investigated the functional responses associated with retaliatory killing and recreational sport hunting of leopards in the Waterberg District Municipality (WDM) in the Limpopo Province of South Africa. We specifically tested the prediction that retaliatory killing will follow a specialist strategy, which could have destabilizing demographic effects, and that recreational sport hunting will follow a generalist predation strategy with subsequent stabilizing demographic effects. We use leopards as a model species as they are a highly adaptable generalist large felid with a wide habitat tolerance which commonly occur on non-protected areas throughout its range [25]. Leopards share many of the characteristics of other large carnivores, e.g. they are frequently in conflict with livestock and game farmers, they are a sought after species in the recreational sport hunting industry [3,26] and they normally occur at low densities on non-protected areas [27].

## Materials and Methods

### Ethics statement

Since our field work consisted of camera trapping and subsequently did not involve any handling of animals, no ethical clearance was required for our surveys. Furthermore, all our study sites were privately owned and we did not need permits from provincial authorities in South Africa to conduct surveys on these sites. We had permission from all private reserves, as well as private landowners to conduct camera trapping on their properties. Future permissions for each study site can be obtained from the appropriate reserve management (www.lapalala.com; www.welgevonden.org) or the Waterberg Biosphere (www.waterbergbiosphere.org) for the farming matrix. The leopard is classified as ‘Near Threatened’ by IUCN [25] and no animals were physically captured or removed from the study sites.

### Study area

**Fig 1:**
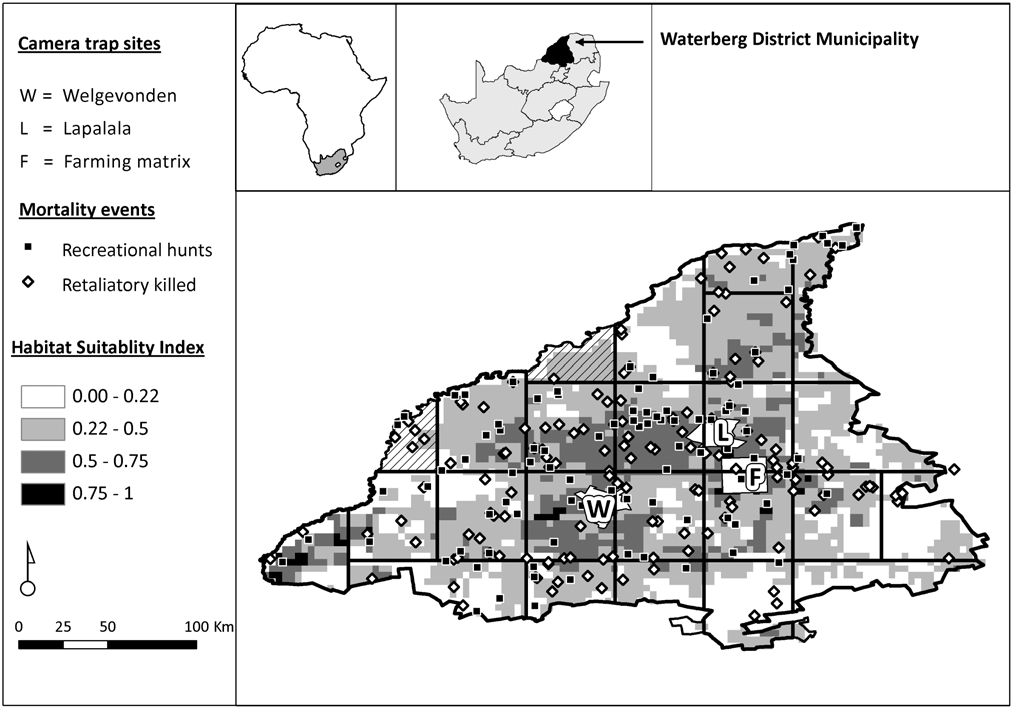
Predicted probability of leopard presence as estimated by a MaxEnt model, distribution of leopards killed in retaliatory incidents, leopard trophy hunts and camera trapping study sites in the Waterberg District, Limpopo Province, South Africa. Striped grids represent grid blocks excluded from analysis because sampling area was below our minimum sample area criteria. MaxEnt model taken from Swanepoel et al. [32].

The Waterberg District Municipality (WDM) is situated in the Limpopo Province of South Africa and covers an area of approximately 49 726 km^2^ (Fig 1). Limpopo Province is the most important province for leopard recreational sport hunting in South Africa, averaging a trophy harvest of 32 leopards per year for the 2000–2010 period (63% of all trophy hunted leopards [28]). Within the WDM the game farming and hunting industry is particularly well established with an estimated 1707 registered game farms (26% of municipal land area [29,30]). The majority of leopard trophy hunts in Limpopo Province also occurs within the WDM (55%; Swanepoel, unpublished), making the WDM an ideal study site to investigate the response of human hunters to varying leopard abundances. Vegetation of the WDM lies within the Central Bushveld bioregion of the Savanna biome [31]. Mining, agriculture and eco-tourism are the main economic activities [29].

### Estimating leopard abundance

We used the leopard density estimated from camera trapping in conjunction with a previously published leopard habitat suitability model [32] to generate spatially explicit leopard abundances [33]. Camera trapping was done at three different sites in the WDM (Fig 1); Lapalala Wilderness (360 km^2^; 23°44´-23°57´S, 28°09´-28°25´E), Welgevonden game reserve (375 km^2^; 24°10´-24°25´S, 27°45´-27°56´E) and a matrix of agricultural farms (350 km^2^; 28°23´E, 24°64´S). For camera trapping we followed guidelines for closed mark recapture studies [34], where we divided each site into 45–65 grid cells each measuring 6.25 km^2^. Our choice in grid size (6.25 km^2^) was motivated by the size of the smallest leopard home range recorded in mountainous terrain (10 km^2^ [35]), which allowed deployment of two camera trap pairs in a home range [34]. Due to a shortage of camera traps we surveyed between 13–15 grid cells for 18–21 days, before moving to the next 13–15 grid cells until completion of each site [36]. Camera trapping was done during the dry season (May 2009 until September 2009) and total time survey time per site ranged from 40 to 90 days, which satisfied assumptions of demographic population closure [36]. At Lapalala and the agricultural farms we used a combination of film and digital camera traps and at Welgevonden only digital infrared camera traps. Leopards were identified by examining spots on flanks and limbs, sex and age identified based on external genitalia, size of neck and general size [26] and all pictures were stored on an image database [37]. Further detailed description of the camera trapping protocol can be found in Swanepoel [38].

Leopard density was estimated from the camera trapping data by fitting maximum likelihood based spatially explicit capture recapture (SECR) models to the capture data [39]. SECR models estimate the home range (activity center) and density of animals directly, which is advantages over traditional buffer strip methods [39,40]. SECR models make the explicit assumptions that the activity centre is fixed during the study, that home ranges are circular and that encounter rate decreases with increased distance from home ranges [41]. The half-normal detection function is normally used to determine the relationship between the detection of an animal and its home range. The detection function has two parameters, sigma (σ) which is a scale parameter describing the decline in encounter rates as distance from home range center increases and an encounter rate (g0) parameter describing animal detection at the home range center [41]. The sex of large carnivores affects both sigma and the encounter rate, and as such density estimates [40,42]. Therefore, we estimated leopard density by fitting a half-normal detection function by maximising the conditional likelihood in which the scale parameter (σ) and the detection probability (g0) were allowed to vary by the sex of the animal [42].

The leopard habitat suitability model is based on presence only modelling using maximum entropy algorithms [32,43]. We choose this maximum entropy method since it is robust in estimating species distributions when data are scare [44,45]. A complete description of the method and the specific habitat suitability model can be found in Swanepoel et al. [32]. The model has a resolution of 10 km^2^. We calculated the average logistic suitability score for each of the three study sites, and extrapolated these abundances across the whole Waterberg in a two-step process. First, we estimated the expected abundance in a 10 km^2^ pixel with ideal habitat (i.e. with a logistic score of 1) *A* by calculating the average across the three study sites:

$$A=\frac{1}{K}\overset{K}{\underset{i=1}{\sum }}\left(\frac{1}{ni}\overset{ni}{\underset{j=1}{\sum }}\frac{a_{ij}}{hs_{ij}}\right)$$ (1)

Where K is the number of study sites (i.e. 3), n_*i*_ is the total number of 10 km^2^ pixels in respective site, and a_*ij*_ and hs_*ij*_ is the estimated abundance (taken from camera trapping results) and logistic habitat suitability score for the *j*th 10 km^2^ pixel in the *i*th study site. We then used this expected maximum abundance to calculate the expected abundance in each pixel by multiplying it with the logistic habitat suitability score:

a'i=A×hsj (2)

Since a’ gives an estimate of the expected number of leopards in each 10 km^2^ pixel, the number of leopards in any given area can be estimated by summing the a’ values of the pixels in the area of interest.

### Estimating predation rates on leopards

As a CITES appendix I species, a permit needs to be issued for each leopard killed as a damage-causing animal (DCA) or through recreational sport hunting. The South African authorities did not have reliable data on whether or not an issued DCA permit resulted in the death of the leopard. However, a large number of leopards in South Africa are killed without DCA permits [28]. We therefore believe that if landowners go to the trouble to apply for a permit, they are likely to kill the leopard. For this analysis we therefore assumed that each DCA permit resulted in the death of a leopard, and we regarded this death as the result of retaliatory killing. We collected data on the number of leopards killed either through retaliatory actions or recreational sport hunting from the provincial nature conservation authority. For each leopard we extracted the date and spatial location (e.g. farm or reserve name; median size 1868 ha). Our analysis was restricted to the period 2002–2010 (9 years). In total, the data included 200 animals killed in retaliatory actions and 133 recreational sport hunted animals with known location data. A detailed description of the leopard mortality data can be found in Swanepoel et al. [46].

### Estimating functional responses

To allow comparisons between local abundances and numbers of animals killed, we divided the study area into a grid consisting of 30 cells comprising 2,500 km^2^ each. However, some cells at the periphery of the study areas were cropped and did not reach this size. Since some of these cropped cells were small, we combined border cells so that no cell included in the analyses was smaller than half the complete size (i.e. 1250 km^2^), to avoid bias in sampling small spatial cells. We choose a grid resolution of 30 cells, since this approximates the cell size of the administrative units for both problem animal and trophy hunting permits (average size of 2650km^2^).

We estimated the number of leopards killed through retaliatory actions or during recreational sport hunting by counting the number of killed animals in each cell. We estimated the leopard abundance in corresponding cells by summing the expected abundance of the containing pixels as described above. For cells that were smaller than the full size, we calculated the corresponding abundance and number of animals killed that corresponded to a full cell size to facilitate easy interpretation of the results.

We tested three functional response models [47,48], representing a type I functional response (linear model), a type II functional response (a hyperbolic model) and a type III functional response (sigmoidal model), each defined as:

TypeI:y=a+b×abundance (3)

TypeII:y=(k×abundance)(x+abundance) (4)

TypeIII:y=(k×abundancer)(xr+abundancer) (5)

where *y* is the number of leopards killed, *b* is the change in number of leopards killed per change in abundance, *k* is the asymptotic abundance at which killing is saturated, *x* is the abundance associated with *k*/2, and *r* is a learning parameter which is associated with the degree to which predators recognise and react to changes in prey abundance [49]. The type II and type III equations are re-formulations based on the Michealis-Menton function which allow type II and type III responses without including time constraints needed for Hollings [13] original equations [49]. We used sample size corrected Akaike information criteria (AICc) to select the most parsimonious model and regarded models within two AICc units as having equal support [50,51]. After each regression we validated that the model assumptions were met using standard criteria, including checking for outliers. Statistical analysis was done in R version 2.15.1 [52] and the user contributed package ‘minpack.lm’ was used to fit the models to the data using the Levenberg-Marquardt algorithm [53], and the user contributed package ‘nlstools’ was used to assess quality of fit of nonlinear models [54].

## Results

### Leopard abundance

We identified 12 individual leopards at Lapalala (7 males, 5 females), 18 at Welgevonden (5 males, 13 females) and 12 in the farming matrix (6 males, 6 females). Leopard density estimates based on the spatially explicit capture recapture model were 5.17 individuals/100 km^2^ ± SE 2.8 for Lapalala, 4.56/100 km^2^ ± 1.3 for Welgevonden and 8.95/100 km^2^ ± 7.7 for the matrix of agricultural farms. The expected abundance at a logistic habitat suitability of 1 was 1.06 (±0.16). We estimated total leopard population in the Waterberg District to consist of 1752 animals.

### Functional responses

**Fig 2:**
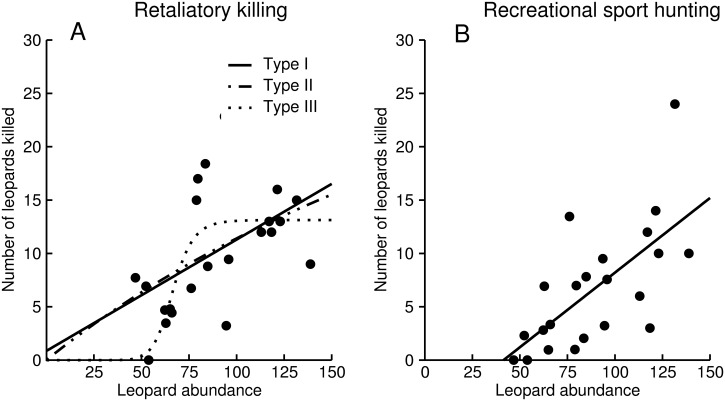
Relationships between leopard abundance and number of animals killed in retaliatory killing (200 leopards; A) and recreational sport hunting (133 leopards; B) in the Waterberg District Municipality, Limpopo Province, South Africa. Number of animals killed and abundance represent number of animals within one 2500 km^2^ sample unit.

For retaliatory killing, we could not distinguish between a type I (AICc = 138.77), a type II (AICc = 138.45), or a type III functional response (AICc = 137.92; Table 1). For recreational sport hunting we could not distinguish between a type II (AICc = 135.66) or a type III (AICc = 135.02) response, but both had lower support compared to a type I response (AICc = 132.41; Table 1). There was a weaker relationship between the number of killed animals and abundance for retaliatory killing compared to recreational sport hunting, leading to higher level of killing at low abundances for animals killed through retaliatory actions compared to recreational sport hunting (Fig 2).

**Table 1:** Akaike information criteria corrected for small sample sizes (AICc), delta AICc (representing the difference in AICc between the current and the most appropriate model) and parameter estimates associated with type I, type II and type III functional response models for leopards killed in retaliatory incidents and leopards hunted and in the Waterberg District Municipality, Limpopo Province, South Africa.

Dependant variable	Type	Parameters			AICc	delta AICc
Retaliatory killing	I	a^1^ = 0.86* ± 3.75	b^2^ = 0.10 ± 0.04		138.77	0.85
	II	k^3^ = 55.56*± 106.07	x^4^ = 387.14* ± 929.72		138.45	0.54
	III	k = 13.13 ± 1.59	x = 66.63 ± 4.46	r^5^ = 12.44*± 9.87	137.91	0
Recreational sport hunting	I	a = - 5.79* ± 3.25	b = 0.14 ± 0.03		132.41	0
	II	k = 8.05E+04* ± 4.33E+08	x = 1.00E+06* ± 5.39E+10		135.66	3.24
	III	k = 8.07E+04* ± 1.09E+09	x = 1.18E+04* ± 8.25E+07	r = 1.94*± 2.62	135.02	2.61

*non-significant parameter at α = 0.05

^1^a is intercept

^2^b is the change in number of leopards killed per change in abundance

^3^k is the asymptotic abundance at which killing is saturated

^4^x is the abundance associated with k/2

^5^r is a learning parameter associated with the degree to which predators recognise and react to changes in prey abundance [49]

## Discussion

Our results suggest that humans engaged in retaliatory killing of leopards responded differently to varying leopard abundance than recreational sport hunters. For retaliatory killing, we could not distinguish between the functional response types, whereas we found unequivocal support for a type I (i.e. a linear response) for recreational sport hunting. These differences resulted in higher levels of retaliatory killing compared to recreational sport hunting, especially at low abundances. We suggest that these differences could lead to different demographic impacts on leopard populations for two reasons. First, more leopards appear to have been killed in retaliatory actions than through recreational sport hunting at similar abundances. This observation alone suggests a higher demographic impact of retaliatory killing. Second, the difference in kill rates between retaliatory killing and recreational sport hunting was progressively higher at lower leopard abundances.

Although we could not find quantitative support for any functional response for retaliatory killing, high levels of predation at low abundances suggest a specialist predation strategy. We provide two reasons why we believe that such a functional response is most appropiate for retaliatory killing of leopards. First, a type I functional response requires consumers to have negligibly small handling time and therefore search for prey at maximum rate and effort [16], which is unlikley retaliatory killing. Secondly, retaliatory killing of carnivores still occur at low carnivore abundances which is indicative of specialist predators [55]. Such specialist predation strategies have been suggested as destabilizing for prey populations [12], and we suggest that retaliatory killing has the potential to be particularly destabilizing since landusers typically are resident, and therefore have the ability to suppress leopard populations to very low numbers if predation is maintained [10,11]. Our lack of ability to distinguish between the three different functional responses could be caused by a number of factors, not mutually exclusive. For instance, it could be caused by a weak hyperbolic relationship which could be difficult to distinguish it from a linear one (e.g., [16]), or our sample size or resolution may not have been sufficient, particularly at low abundances. It is also plausible that multiple permits were issued before a leopard was killed (e.g. difficulty in finding and killing a leopard at low densities) which might have overestimated the number of leopard killed at low abundances.

In contrast, the opportunistic strategy of recreational sport hunters suggests a density dependent harvest, which could be partially stabilizing to the leopard population [18]. These results conform to previous studies on deer (*Odocoileus virginianus*) hunters [24], recreational anglers [56] and recreational lobster divers (*Panulirus argus*; [18]), where exploitation increased linearly with prey abundance. These studies indicate that hunters act opportunistically, but engage in hunting even at low abundances, which suggests that to a certain degree hunters’ willingness to hunt is unresponsive towards the abundance of their potential target. This agrees with the notion that hunters derive rewards from the hunting activity that are only loosely related to hunting success rate [22]. However, we highlight that the lack of support for a type III functional response for recreational sport hunters could have been caused by the spatial scale of our analysis, where our spatial units may not have been small enough to result in low hunting at low abundances [16]. Nonetheless, our results suggest that recreational sport hunters have more complex and diverse motivations than a predator whose fitness depends on its prey.

We did not find an asymptote in number of killed animals for neither retaliatory killing nor recreational sport hunting. Theoretically, an asymptotic functional response arises when there is saturation in killing rate, due to handling time constraints [13,16]. For retaliatory killing, an asymptote would either suggest handling time constraints in killing problem animals [13] or it may indicate saturation in the psychological needs to persecute [19]. However, land users can reduce their handling time constraints by hiring additional staff to set traps and kill leopards. Saturation in the psychological motivation to kills leopards would probably be unrelated to leopard abundance [19]. Therefore, an asymptote in retaliatory killing is likely to be very high. For recreational sport hunting an asymptote could arise due to market saturation, maximum hunting quotas being reached, a limitation in the number of professional outfitters to accompany hunters or a limitation in available hunting areas. However, demand for leopard trophies has been increasing [26], the hunting quota in South Africa has been doubled [57], and the study area has highly suitable leopard habitat [32] and has a well-established hunting industry [29]. Therefore conditions that may induce an asymptote in trophy hunting do not seem to be currently limited in our study area, which can explain the lack of an asymptote.

## Policy Implications

Our results from this study support previous research from stochastic simulation models [46] and suggest that retaliatory killing of leopards may have more severe demographic consequences for leopards compared to trophy hunting. We therefore emphasize the importance of including retaliatory killing in the evaluation of population viability and setting of harvest quotas [40]. We also highlight the importance of developing and implementing policies focused on non-lethal conflict mitigation. Such non-lethal mitigation strategies can include livestock guarding dogs [20], kraaling of livestock [58] or various compensation schemes [59]. We suggest that such policies will probably be more efficient than harvest restrictions alone in improving the viability of demographically threatened leopard populations.
